# Book Review: Oxidative Damage to Plants-Antioxidant Networks and Signaling

**DOI:** 10.3389/fpls.2015.00452

**Published:** 2015-06-17

**Authors:** Naser A. Anjum

**Affiliations:** Department of Chemistry, CESAM-Centre for Environmental and Marine Studies, University of AveiroAveiro, Portugal

**Keywords:** abiotic stress, Reactive Oxygen Species, Oxidative Stress, Antioxidant networks, ROS signaling

Plants, having sessile nature have to interact with immediate environment for sustaining their lives. Since biotic and abiotic stresses are dominating the favorable growth conditions, plants have to deal with their impact (such as oxidative stress: an imbalance between production and metabolisms of reactive oxygen species, ROS), and maintain their genetic potential to maximally grow and produce (Anjum et al., 2010, 2012, 2014). Notably, considering their direct or indirect involvement in making food, fuel, fiber, and shelter available to human, understanding intricacies of and improving plant life processes under sub-optimal conditions seems timely and imperative.

The entitled above edited volume comprising 20 chapters contributed by eminent researchers, is an attempt to gain insights into both the devastating and signaling role of oxidative stress, and the major defense strategies adopted in plants.

Focused at “ROS and photosynthesis,” the Chapter 1 introduces main steps of light and dark reactions, and discusses the production and scavenging of excess excitation energy (EEE) and ROS since their significant role in photosynthesis under environmental stresses. Besides, before briefly discussing the evolution of plant adaptation mechanisms to counteract the damaging effects of EEE and ROS, the author nicely presented the facts how photosynthesis can be regulated by redox status and ROS through their contribution in the signaling pathways. ROS have been implicated as second messengers in plant hormone responses. To this end, the discussion outcomes of the Chapter 2 unveiled that an integrated network, formed by ROS and plant hormone signaling pathways involves a number of crosstalk pathways (related with roles of ABA, ethylene, JA, and MeJA), and can regulate plant growth, development, and their responses to environmental factors.

Discussion in the Chapter 3 is centered on the superoxide dismutase (SOD), the first line of defense in plants against varied ROS such as superoxide (O^·−^_2_) that is generated after the reduction of molecular O_2_ and is considered to have strong reactivity and oxidizing ability. The author has thoroughly reviewed the significance and modulation of SOD in abiotic stressed plants. The dismutation of O^·−^_2_ by SOD yields H_2_O_2_, a non-radical and longer half-time (1 ms) having ROS. H_2_O_2_ has the capacity to diffuse and reach numerous biomolecules and affect the activity of proteins oxidizing the thiol groups. Hence, considering the role of H_2_O_2_ in plants, and the availability of very little information on the versatility of catalase (CAT) in plants, the Chapter 4 presents a thorough review of the overall aspects of CAT in plants, where CAT, with its excellent enzyme kinetics efficiently remove H_2_O_2_ without needing any reducing substrate.

This book presents good reviews on the significance, modulation and metabolism of both major enzymatic and non-enzymatic antioxidants in abiotic stressed plants. In this context, centered mainly on a major non-enzymatic antioxidant–glutathione (GSH), the Chapters 5 and 6 discuss GSH modulation, metabolism and role in cellular redox homeostasis-maintenance in abiotic stressed plants. However, in Chapters 7–10, various aspects of the other major non-enzymatic antioxidants, such as ascorbic acid, carotenoids, and lipophylic molecules are discussed in plants exposed different stress conditions. The generation, sites, status and modulation of varied ROS, their impact, control, and transcriptional regulation are critically discussed in different plant species exposed to major abiotic (drought, Chapter 11; temperature, Chapter 12) and biotic (pathogen and wounding, Chapter 13) stresses. Major focus of Chapter 14 is on the role of ascorbate peroxidase (APX) in postharvest treatments for horticultural crops. It was suggested that shelf life can be improved and quality can be retained during postharvest handling of horticultural crops if efforts are made to modulate or control abiotic stresses in plant tissues.

Role of mycorrhizas in the control of ROS and their impact via the modulation of various enzymatic and non-enzymatic components in plants was summarized in the Chapter 15. Major biochemical and molecular mechanisms underlying the various roles played by iminoacid–proline are thoroughly discussed in the Chapter 16; whereas, the Chapter 17 highlights the modulation and control of plant tolerance to trace elements by various components of plant antioxidant system. Various aspects related with their generation, role and signaling of ROS in plants are the major focus of the Chapters 18–20. Nevertheless, the large scale production of stress tolerant plants can be possible with the clear understanding of the role of signaling in plants under stresses. These aspects were briefly dealt particularly in the Chapter 18; whereas, insights into the generation, scavenging and signaling of various ROS including H_2_O_2_ in plants were nicely presented in the Chapters 19 and 20.

The book is not well-structured, and the contents are not logically arranged. It would have been more imperative if the editor has arranged the whole content of this book as follows: (1) the generation, sites, status and modulation, impact and signaling of varied ROS in plants under sub-sections 1A and 1B for abiotic and biotic tresses respectively, and (2) ROS status and its control via enzymatic (2A), and non-enzymatic (2B) components of plant defense system. Additionally, this book has considered only two enzymes (CAT and APX) as major ROS/H_2_O_2_ scavengers in plants, and has given very least importance to the role of ROS, oxidative stress, signaling and control in plants under major biotic stresses.

The overall coverage of the subject matter in the present edited volume is satisfactory, where major aspects related with ROS, oxidative stress and their control via antioxidant networks and signaling were discussed. Thus, the present edited volume may be a good asset for young scientists and master students in plant science area.

This book can be found at: http://www.elsevier.com/books/oxidative-damage-to-plants/ahmad/978-0-12-799963-0

## Conflict of interest statement

The author declares that the research was conducted in the absence of any commercial or financial relationships that could be construed as a potential conflict of interest.
